# Correlation among baseline variables yields non-uniformity of p-values

**DOI:** 10.1371/journal.pone.0184531

**Published:** 2017-09-08

**Authors:** Rebecca A. Betensky, Sy Han Chiou

**Affiliations:** Department of Biostatistics, Harvard T.H. Chan School of Public Health, Boston, Massachusetts, United States of America; Texas A&M University, UNITED STATES

## Abstract

A recent paper in *Neurology* used statistical techniques to investigate the integrity of the randomization in 33 clinical trials conducted by a group of investigators. Without justification, the approach assumed that there would be no impact of correlation among baseline variables. We investigated the impact of correlation on the conclusions of the approach in several large-scale simulation studies that replicated the sample sizes and baseline variables of the clinical trials in question and utilized proper randomization. Additionally, we considered scenarios with larger numbers of baseline variables. We found that, with even moderate correlation, there can be substantial inflation of the type I error of statistical tests of randomization integrity. This is also the case under no correlation, in the presence of some discrete baseline variables, with a large number of variables. Thus, statistical techniques for assessing randomization integrity should be applied with extreme caution given that very low p-values, which are taken as evidence against valid randomization, can arise even in the case of valid randomization, in the presence of correlation. More generally, the use of tests of goodness of fit to uniformity for the purpose of testing a global null hypothesis is not advisable in the presence of correlation.

## Introduction

A recent paper by Bolland et al. [1] examined the integrity of the randomization of a set of 33 clinical trials that were conducted and published by a set of researchers over a 15 year period. There were several parts to their analysis and the ultimate finding of fraud is not in question as the lead researcher admitted to it and retracted some publications, e.g. Gross [2]. In this article, we focus on one aspect of the statistical analysis in Bolland et al. [1]: their assessment of the uniformity of the p-values comparing baseline characteristics between randomized treatment groups. This analysis hinges on the theoretical result that under the null hypothesis, p-values are uniformly distributed, and so significant deviation from uniformity across baseline variables and across trials would be evidence against the null hypothesis of random allocation to treatment groups. The authors recognized that this theoretical result assumes independence of the p-values, which would not be a reasonable assumption for subsets of baseline variables from clinical trials, such as blood pressure, cholesterol, age, BMI, etc. However, they dismissed this as unimportant as long as the correlation is consistent across trials. Even assuming consistency of correlation, itself a questionable assumption as populations of participants within each trial may be slightly different, the authors did not provide any evidence for their conclusion. In fact, it contradicted the limited simulation findings of Bland [3], though these were not directly applicable to the Bolland et al. [1] analysis. Thus, we extensively investigated its validity using the features of the group of clinical trials in question.

It is a basic theoretical result that a p-value for a continuous random variable that is calculated using the true distribution of the random variable follows a standard uniform distribution. This is not true when the p-value is calculated using an incorrect reference distribution. For example, a p-value based on a t-test comparing two samples of log-normally distributed random variables does not follow a uniform distribution. This result does not hold for discrete distributions; a p-value from Fisher’s exact test or a chi square test to compare two groups of binary data under the null does not follow a uniform distribution. Extending this result to a sample of p-values, it is clear that if an individual p-value is not uniformly distributed for reasons given above, a sample of these p-values will likewise not represent a sample from a uniform distribution. Additionally, even if individually the p-values follow a uniform distribution, a sample of correlated p-values may not behave like a random sample from a uniform distribution.

These issues were discussed by Bland [3], who conducted limited simulations to illustrate these points. In a few simulations of the deviations from the requirements for uniformity of p-values, he assessed the uniformity of the p-values through visual inspection. His conclusion was that due to the likely deviations from these requirements, the concept of assessing p-values from baseline comparisons between randomized groups in order to verify proper randomization does not work under many circumstances.

In spite of the cautionary message in Bland [3], Bolland et al. [1] used this method in their analysis of the 33 clinical trials conducted by one group of researchers. In particular, the Bolland et al. [1] analysis examined 33 clinical trials: 31 were two group comparisons and 2 included three groups. The sample sizes for the trials are in Table 1 of Bolland et al. [1]. There were approximately 17 baseline variables measured in each trial, of which approximately 13 were continuous. T tests were used for two-group comparisons of continuous data and Fisher’s exact tests were used for binary data. From the amalgamation of 513 p-values, 52% were greater than 0.8, 6% were less than 0.2, 14% were less than 0.4, and 27% were less than 0.6; the chi square p-value for comparing this observed distribution to the expected under uniformity is reported to be 5.2×10^−82^. This goodness of fit test does not account for within-person correlation of the baseline variables, and thus of the associated within-trial p-values.

**Table 1:** Rejection proportions from 100,000 repetitions of each simulation scenario: 500 variables (100 Bernoulli variables, with p’s drawn from Uniform(0.2,0.8) and fixed across all repetitions).

	Model 1		Model 2		Model 3	
	chi square test	KS test	chi square test	KS test	chi square test	KS test
**rho: fixed**^1^						
0	0.149	0.622	0.149	0.620	0.049	0.049
0.1	0.307	0.625	0.332	0.634	0.258	0.298
0.2	0.513	0.714	0.552	0.732	0.532	0.569
0.3	0.646	0.794	0.685	0.812	0.680	0.709
0.4	0.742	0.852	0.772	0.866	0.775	0.798
0.5	0.821	0.897	0.843	0.909	0.850	0.869
Uniform(0.4,0.9)	0.943	0.967	0.950	0.971	0.961	0.972
**rho: random**^2^						
Uniform(0.4,0.9)	0.945	0.968	0.953	0.972	0.963	0.973

^1^ rho is fixed for each clinical trial in all repetitions of these simulations;

^2^ rho is randomly generated from Uniform (0.4,0.9) for each clinical trial in each repetition of the simulation

Because the simulation results of Bland [3] were limited to one or two variables and did not involve evaluation of formal tests of uniformity, we undertook a comprehensive simulation study that used the structure of the Bolland et al. [1] analysis. In particular, we used the sample sizes from those trials, the numbers of continuous and binary variables measured at baseline, and we considered a range of correlation structures among variables and across trials. We conducted all of our simulations under the null hypothesis of valid randomization. That is, we generated data from the same distributions for the two groups in each trial. Additionally, we considered larger numbers of variables than were available from the Bolland et al. [1] collection of trials to learn more generally about the effects of correlation on uniformity of null p-values.

## Materials and methods

We considered three experimental settings: in the first we included 500 baseline variables with either 100 or zero binary variables, in the second we included 17 baseline variables with either four or zero binary variables, and in the third we included 60 baseline variables with either 14 or zero binary variables. The second setting corresponds to the trials in Bolland et al [1]. In all of our simulation scenarios we generated the continuous baseline variables from equally correlated, mean zero, variance one, multivariate normal distributions. We simulated the correlation, rho, in two manners: in the first, the 31 values of rho—one for each clinical trial—were pre-assigned, and held fixed for each repetition of the simulation, and in the second, the 31 values of rho were generated anew in each repetition. In particular, we assigned a correlation for the baseline variables for each clinical trial using four different approaches: (a) rho fixed across all repetitions of the simulation and the same for each clinical trial (as assumed by Bolland et al. [1] to be consistent with uniformity of p-values), with rho = 0, 0.1, 0.2, 0.3, 0.4, 0.5; (b) rho fixed across all repetitions of the simulation, with the 31 rho’s drawn independently from a Uniform(0.4,0.9); (c) rho variable across all repetitions of the simulation, with the 31 rho’s drawn independently from Uniform(0.4,0.9) at each repetition. The latter two correlation models address the possibility that there may be variation in the clustering of variables across trials, as noted by Bolland et al [1]. R code for implementation of our simulation studies is available in S1 File.

In all of our simulations, we fixed the “success” probabilities, p, associated with each of the binary baseline variables, to be the same for each clinical trial and for each repetition of the simulation. We generated these probabilities from a Uniform(0.2,0.8) for our simulations that included 100 or 15 binary variables, and we set them to be (0.2,0.4,0.6,0.8) for the simulation that included four binary variables. The effect of different probabilities for the binary variables is on the power of the tests comparing the randomized groups and on the behavior of the associated p-values.

Within each repetition of our simulation, we generated baseline data from 31 clinical trials of the same sample sizes as in [1] according to the following models, where N is the total number of baseline variables (i.e., 500, 17 or 60) and B is the total number of binary baseline variables (i.e., 100, 4 or 15):

1. Model 1: We generated N-B baseline variables that were normally distributed, mean 0, variance 1, and correlation rho, and we generated independent B binary variables using the generated binary p’s.
2. Model 2: We generated N baseline variables that were normally distributed, mean 0, variance 1, and correlation rho, and dichotomized B of them according to the normal quantile associated with the binary p for that variable.
3. Model 3: We generated N baseline variables that were normally distributed, mean 0, variance 1, and correlation rho.

Note that all N variables are mutually correlated in Models 2 and 3, while in Model 1 only the N-B normally distributed variables are mutually correlated and the remaining B binary variables are independent of each other and of the N-B normal variables. We calculated 31×N p-values comparing the two randomized groups for each of the N variables, for each of the 31 trials. We used t-tests for the normally distributed random variables and Fisher’s exact tests for the binary variables. We then applied two goodness of fit tests to this collection of p-values: (1) the multinomial goodness-of-fit test with continuity correction (chisq.test in R) to compare the observed proportions of p-values within the intervals (0,.2), (0.2,0.4), (0.4,0.6), (0.6,0.8) and (0.8,1.0) to the expected proportion of 20% for each based on a uniform distribution; and (2) the Kolmogorov Smirnov (KS) goodness of fit test (ks.test in R) to test uniformity of the p-values. We repeated these simulations 100,000 times and calculated the proportion of repetitions in which the goodness of fit test p-values were less than 0.05.

## Results

Tables 1–3 list the results of our simulation study, corresponding to (N,B) = (500,100), (17,4), and (60,15), respectively. If p-values under the null hypothesis of valid randomization (i.e., no difference between groups for any of the N variables) were uniformly distributed, we would expect both the multinomial test and the KS test to yield rejection proportions of 0.05. For any number of variables (500, 17, 60), this is the case when rho = 0 and when there are no binary variables included (Model 3). When 100 binary variables are included among the 500 total variables (Table 1, Models 1 and 2), the rejection proportion of the multinomial test is 0.149 and that of the KS test is approximately 0.62. This indicates that uniformity of p-values under the null is not achieved even when only 20% of those p-values include those from Fisher’s exact test for binary random variables. The rejection proportions for the multinomial and KS tests are close to 0.05 when there are 17 variables (Table 2, Models 1 and 2) and are slightly larger (0.08–0.09) when there are 60 variables (Table 3, Models 1 and 2). Thus, if there were no correlation among the 17 baseline variables in the 31 clinical trials of Bolland et al. [1], the p-values from the multinomial test of p-values for testing equality of randomized groups would be approximately valid. In other settings with larger numbers of variables, the type I error is not preserved.

**Table 2:** Rejection proportions from 100,000 repetitions of each simulation scenario: 17 variables (4 Bernoulli variables with p’s equal to 0.2, 0.4, 0.6, 0.8, and fixed across all repetitions).

	Model 1		Model 2		Model 3	
	chi square test	KS test	chi square test	KS test	chi square test	KS test
**rho: fixed**^1^						
0	0.056	0.048	0.056	0.048	0.050	0.048
0.1	0.061	0.055	0.062	0.056	0.056	0.057
0.2	0.072	0.071	0.078	0.079	0.076	0.085
0.3	0.092	0.102	0.107	0.116	0.115	0.133
0.4	0.130	0.146	0.153	0.175	0.172	0.200
0.5	0.175	0.199	0.211	0.238	0.241	0.278
Uniform(0.4,0.9)	0.311	0.343	0.356	0.387	0.417	0.451
**rho: random**^2^						
Uniform(0.4,0.9)	0.305	0.337	0.358	0.388	0.408	0.444

^1^ rho is fixed for each clinical trial in all repetitions of these simulations;

^2^ rho is randomly generated from Uniform (0.4,0.9) for each clinical trial in each repetition of the simulation;

**Table 3:** Rejection proportions from 100,000 repetitions of each simulation scenario: 60 variables (15 Bernoulli variables, with p’s drawn from Uniform (0.2,0.8), and fixed across all repetitions).

	Model 1		Model 2		Model 3	
	chi square test	KS test	chi square test	KS test	chi square test	KS test
**rho: fixed**^1^						
0	0.078	0.089	0.078	0.089	0.049	0.048
0.1	0.082	0.109	0.087	0.116	0.073	0.083
0.2	0.133	0.170	0.155	0.196	0.155	0.184
0.3	0.215	0.264	0.261	0.309	0.276	0.314
0.4	0.313	0.369	0.367	0.419	0.390	0.433
0.5	0.405	0.467	0.467	0.521	0.496	0.535
Uniform(0.4,0.9)	0.620	0.662	0.672	0.708	0.725	0.741
**rho: random**^2^						
Uniform(0.4,0.9)	0.612	0.655	0.663	0.701	0.715	0.733

^1^ rho is fixed for each clinical trial in all repetitions of these simulations

^2^ rho is randomly generated from Uniform (0.4,0.9) for each clinical trial in each repetition of the simulation

Under the important and realistic scenario of correlation among baseline variables, as we would expect in clinical trials, the type I error for the test of uniformity of p-values (i.e., for the test of valid randomization) can be quite inflated. In the case of 500 variables (Table 1), the rejection proportion is greater than 0.95 when some trials have high correlation, and it is over 0.77 when all trials have correlation of 0.4 (Model 2). In the case of 17 variables (Table 2), the rejection proportion is over 0.36 when some trials have high correlation and it is over 0.15 when all trials have correlation of 0.4 (Model 2). In the case of 60 variables (Table 3), the comparable (Model 2) rejection proportions are 0.67 and 0.37. The rejection proportions for Model 1 are generally slightly lower than those for Model 2; this is because Model 1 features correlation only among the continuous variables, while Model 2 features correlation across all continuous and binary variables. The rejection proportions for the KS test for Model 3 are lower than those for Models 1 and 2 due to its consideration of continuous variables only. This is not seen for the chi square test, due to its grouping of p-values and thus lower sensitivity to the Fishers exact p-values from the binary variables.

## Discussion

Bolland et al. [1] tested the validity of the randomization in 33 clinical trials published by a single group of authors by testing whether the p-values from the comparison of baseline variables from randomized groups were uniformly distributed. Bolland et al. [1] claimed that correlation among the baseline variables would not affect this expected result if the correlation were consistent across trials. Bolland et al. [1] did not acknowledge that the presence of binary variables among the baseline variables could affect this expected result, as could application of tests whose assumptions were not met by the data. These latter two points were studied by Bland [3], who showed that even under the null of equality of randomized groups, p-values based on discrete random variables and p-values from t-tests applied to log-normal random variables did not appear to be uniformly distributed. In this paper, we investigated the claim of Bolland et al. [1] that consistent correlation across trials would not affect uniformity of p-values under the null; this was not considered in the analysis of Bland [3]. We additionally considered the impact of binary variables in our analysis, as well as the impact of the number of p-values being analyzed. Our results have applications beyond clinical trials; they apply to any setting in which there are several experiments, such as genomic arrays from several subjects. They are relevant also given the current emphasis on transparency and reproducibility.

We found that correlation can have a substantial impact on the expected uniformity of p-values under the null hypothesis, for exclusively normally distributed random variables, even when it is completely consistent across experiments, and even when it is small in magnitude. For example, Table 1 indicates that in the setting of 500 variables per experiment and under the null of no group differences, when the correlation is 0.1 for each clinical trial, uniformity of p-values is rejected with probability greater than 0.25. When the correlation varies across experiments and ranges from 0.4 to 0.9, this rejection probability is greater than 0.95. This qualitative result is also seen in the setting of 17 variables per clinical trial, though at higher levels of correlation and with rejection probabilities of up to 0.40.

Based on our simulations, the results of Bolland et al. [1] are reliable only in the case of zero correlation among the 17 baseline variables that were measured in the clinical trials. This is not a plausible scenario. We conclude that such statistical techniques should be applied with extreme caution given that very low p-values, which are taken as evidence against the null of valid randomization, can result even under the null. Our simulation study shows that this happens even in the presence of a low level of correlation that is consistent across trials, and even with no correlation in the presence of binary variables. Given that these results may lead to the impugning of researchers’ reputations, it is essential to thoroughly examine the behavior of such statistical tests in a wide range of plausible settings before employing them to examine data integrity. More generally, we have demonstrated that the use of tests for goodness of fit to uniformity as a means of testing a global null hypothesis can be quite misleading in the presence of correlation among subsets of variables. One possible alternative approach if randomization of a clinical trial were in question would be to test for uniformity one variable at a time, and to correct for multiple testing across the 17 variables.

## Supporting information

S1 File R code for simulations. (PDF)
