# Supplementary material for: Correlation among baseline variables yields non-uniformity of p-values
Source: PLoS One. 2017 Sep 8;12(9):e0184531. doi: 10.1371/journal.pone.0184531 (PMC5590981; doi:10.1371/journal.pone.0184531)
Supplement: S1 File — (PDF) [file pone.0184531.s001.pdf]

Supporting information for “Correlation among baseline variables yields non-uniformity of p-values”

1 R code for simulations

The simulation codes require the following package:

```
library(MASS)
```

The following function provides one simulation run

```
sim <- function(N, rho, p) {
    dat <- NULL
    nBin <- length(p)
    nNorm <- N - nBin
    if (length(rho) != 31) stop("rho length must be 31")
    if (nNorm < 0)
        stop("number of Bernoulli variables must be less than the total number of variables")
    ## Declare case and control size
    nCase <- c(45,54,43,49,60,20,129,40,25,67,314,48,140,132,187,250,
               100,25,41,144,121,61,56,34,40,97,116,136,162,41,26)
    nCont <- c(39,54,43,49,60,20,129,40,25,68,314,48,140,132,187,250,
               100,25,41,144,121,61,56,34,40,97,115,136,162,41,26)
    nTrail <- apply(cbind(nCont, nCase), 1, sum)
    ## Prepare general data.frame
    dat$Trail <- rep(1:31, nTrail)
    dat$case <- do.call(c, sapply(1:31, function(x) c(rep(1, nCase[x]), rep(0, nCont[x]))))
    dat <- data.frame(dat)
    ## First generate N normal variables
    vNorm <- sapply(1:31, function(x)
        mvrnorm(nTrail[x], mu = rep(0, N), Sigma = matrix(rho[x], N, N) + diag(1 - rho[x], N)))
    ## Prepare data
    ## Prepare data for Model 1
    vNorm <- do.call(rbind, vNorm)
    vNorm1 <- vNorm[, 1:nNorm]
    vBin1 <- sapply(1:nBin, function(x) rbinom(sum(nTrail), 1, p[x]))
    dat1 <- cbind(dat, vNorm1, vBin1)
    ## Prepare data for Model 2
    vNorm2 <- vNorm[, 1:nNorm]
    vBin2 <- invisible(sapply((nNorm + 1):N, function(x) 1 * (vNorm[,x] < qnorm(p[x - nNorm]))))
    dat2 <- cbind(dat, vNorm2, vBin2)
    ## Preapre data for Model 3
    dat3 <- cbind(dat, vNorm)
    ## Calculating p-vales
    ## Model 1
    pvalNorm1 <- sapply(1:31, function(x) sapply(3:(nNorm + 2), function(y)
        t.test(subset(dat1, subset = Trail == x & case == 1, select = y, drop = TRUE),
               subset(dat1, subset = Trail == x & case == 0, select = y, drop = TRUE))$p.value))
    pvalBin1 <- sapply(1:31, function(x) sapply((nNorm + 3):(N + 2), function(y)
        fisher.test(rbind(table(subset(dat1, subset = Trail == x & case == 1, select = y)),
                          table(subset(dat1, subset = Trail == x & case == 0, select = y))))$p.value))
    pval1 <- sort(c(pvalNorm1, pvalBin1))
    ## Model 2
    pvalNorm2 <- sapply(1:31, function(x) sapply(3:(nNorm + 2), function(y)
        t.test(subset(dat2, subset = Trail == x & case == 1, select = y, drop = TRUE),
               subset(dat2, subset = Trail == x & case == 0, select = y, drop = TRUE))$p.value))
    pvalBin2 <- sapply(1:31, function(x) sapply((nNorm + 3):(N + 2), function(y)
        fisher.test(rbind(table(subset(dat2, subset = Trail == x & case == 1, select = y)),
                          table(subset(dat2, subset = Trail == x & case == 0, select = y))))$p.value))
    pval2 <- sort(c(pvalNorm2, pvalBin2))
    ## Model 3
    pval3 <- sort(sapply(1:31, function(x) sapply(3:(N + 2), function(y)
        t.test(subset(dat3, subset = Trail == x & case == 1, select = y, drop = TRUE),
               subset(dat3, subset = Trail == x & case == 0, select = y, drop = TRUE))$p.value)))
    ## KS test and chi-square test are used to test for the uniformity of the p-values
    count1 <- diff(sapply(seq(0, 1, .2), function(x) sum(pval1 < x)))
    count2 <- diff(sapply(seq(0, 1, .2), function(x) sum(pval2 < x)))
    count3 <- diff(sapply(seq(0, 1, .2), function(x) sum(pval3 < x)))
    out <- c(ks.test(unique(pval1), punif)$p.value, chisq.test(count1, p = rep(.2, 5))$p.value,
```

```
            ks.test(unique(pval2), punif)$p.value, chisq.test(count2, p = rep(.2, 5))$p.value,
            ks.test(unique(pval3), punif)$p.value, chisq.test(count3, p = rep(.2, 5))$p.value)
    names(out) <- c("M1.KS", "M1.chi", "M2.KS", "M2.chi", "M3.KS", "M3.chi")
    return(out)
}
```

The argument `N` is the total number of baseline variables, consisting of continuous or discrete variables. The argument `rho` is a vector of 31 correlation for the 31 clinical trails. The argument `p` is the success probabilities associated with each of the Bernoulli variables. The length of `p` also equals to the number of Bernoulli variables among the `N` baseline variables. The function generates simulated data under all three models and returns p-values from the uniformity tests. The following example provies one simulation run under the settings in the first row of Table 1

```
## Fix p from a Uniform(0.2, 0.8) across all replications
p0 <- c(0.35931, 0.42327, 0.54371, 0.74492, 0.32101, 0.73903, 0.76681, 0.59648, 0.57747,
        0.23707, 0.32358, 0.30593, 0.61221, 0.43046, 0.66190, 0.49862, 0.63057, 0.79514,
        0.42802, 0.66647, 0.76082, 0.32729, 0.59100, 0.27533, 0.36033, 0.43167, 0.20803,
        0.42943, 0.72181, 0.40421, 0.48925, 0.55974, 0.49612, 0.31173, 0.69642, 0.60108,
        0.67654, 0.26477, 0.63423, 0.44676, 0.69257, 0.58824, 0.66976, 0.53182, 0.51783,
        0.67361, 0.21400, 0.48634, 0.63939, 0.61564, 0.48657, 0.71673, 0.46286, 0.34688,
        0.24241, 0.25968, 0.38976, 0.51118, 0.59720, 0.44410, 0.74773, 0.37616, 0.47544,
        0.39944, 0.59052, 0.35481, 0.48713, 0.65979, 0.25055, 0.72519, 0.40344, 0.70366,
        0.40801, 0.40026, 0.48581, 0.73532, 0.71860, 0.43399, 0.66639, 0.77637, 0.46080,
        0.62751, 0.44000, 0.39521, 0.65425, 0.32162, 0.62667, 0.27302, 0.34729, 0.28598,
        0.34378, 0.23536, 0.58537, 0.72576, 0.66735, 0.67839, 0.47316, 0.44605, 0.68652,
        0.56296)
sim(N = 500, rho = rep(0, 31), p = p0)

     M1.KS     M1.chi      M2.KS     M2.chi      M3.KS     M3.chi
0.01294318 0.27767828 0.02065433 0.09539111 0.72468895 0.68935636
```

To reproduce the results presented in the first row of Table 1, one needs to repeat this process 100,000 times, and compute the rejection proportions accordingly. The following gives an exaple with `replicate`, however, in partice, parallel counting is recommanded.

```
## Not run
## foo <- replicate(100000, sim(N = 500, rho = rep(0, 31), p = p0))
## apply(foo, 1, function(x) sum(x < .05) / 100000)
```

The next example gives one simulation run under the settings in the 7th row of Table 1 where rho is hold fixed at one draw from Uniform(0.4, 0.9).

```
p0 <- .2 * 1:4
rho0 <- c(0.8113537,0.8564772,0.5280694,0.5312012,0.6916341,0.8928680,0.7110176,
          0.7665706,0.8100644,0.8450741,0.4513224,0.4881750,0.4120449,0.5871851,
          0.4136202,0.7394356,0.7760288,0.8964444,0.4955094,0.8186529,0.7054409,
          0.8292236,0.5017718,0.6783677,0.5714878,0.6202944,0.7994758,0.7637531,
          0.8454874,0.5894430,0.8800838)
sim(N = 17, rho = rho0, p = p0)

       M1.KS       M1.chi        M2.KS       M2.chi        M3.KS
1.530759e-02 1.555307e-02 1.889005e-04 2.127154e-03 1.541211e-04
      M3.chi
7.725999e-05
```

The remaining results in the Tables can be reproduced similarly.
